# Intrabullous Adhesion Pexia (IBAP) by Percutaneous Pulmonary Bulla Centesis: An Alternative for the Surgical Treatment of Giant Pulmonary Bulla (GPB)

**DOI:** 10.1155/2018/5806834

**Published:** 2018-10-23

**Authors:** Wei-Liang Li, Yong-Hua Li, Yu-Bo Yang, Li-Hui Lv

**Affiliations:** ^1^Department of Respiratory and Critical Care Medicine, Mingzhou Hospital, Zhejiang University, No. 168 West Taian Road, Ningbo, Zhejiang 315199, China; ^2^Department of Respiratory and Critical Care Medicine, Donghai Hospital, Ningbo University, No. 377 Zhongshan East Road, Ningbo 315040, China

## Abstract

**Background and Objective:**

Most patients with giant pulmonary bulla (GPB) are treated by surgery; however, there is a subset for whom surgery is not a viable option, such as those with contraindications, or those unwilling to undergo operation. Therefore, an alternative minimally invasive method is desired for this subpopulation. The aim of this study was to explore an alternative procedure for treating GPB.

**Methods:**

This was a prospective, nonrandomized, single-arm, unblinded study evaluating the efficacy and safety of intrabulla adhesion pexia (IBAP) procedure in GPB patients. The study was conducted between December 2004 and April 2017.

**Results:**

There were 38 cases in 36 patients (33 males and 3 females) with the target GPB cavities varying in size (range, 10 cm × 7 cm × 5 cm to 15 cm × 8 cm × 30 cm (anteroposterior diameter × medial-lateral diameter × superoinferior diameter)). After IBAP treatment, the closure ratio of GPB in one month was 86.84% (33/38), while the dyspnea index significantly decreased from 4.11 ± 1.11 to 2.24 ± 1.15 (*P* < 0.01). In addition, the mean FEV1 (L) increased from 1.06 ± 0.73 to 1.57 ± 1.13 (*P* < 0.01), while RV (L) decreased from 2.77 ± 0.54 to 2.36 ± 0.38 (*P* < 0.01) and TLC (L) decreased from 6.46 ± 1.21 to 5.86 ± 1.08 (*P* < 0.01). Moreover, PaO_2_ (mmHg) increased from 52.18 ± 8.31 to 68.29 ± 12.34, while the 6 MWD increased by 129.36% from 131.58 ± 105.24 to 301.79 ± 197.90 (*P* < 0.01). Collectively, these data indicated significant improvement in pulmonary function and exercise tolerance after IBAP treatment. Furthermore, no deaths occurred during IBAP treatment, and no cases of aggravated GPB relapse were reported during the 12-month follow-up period.

**Conclusions:**

IBAP is a promising strategy for the treatment of GPB. Our findings demonstrated that IBAP had a noteworthy therapeutic effect, desirable safety, and ideal long-term efficacy for GPB.

## 1. Introduction

Giant pulmonary bulla (GPB) or giant emphysematous bulla (GEB), a bulla occupying more than one-third of the involved hemithorax, usually requires removal by surgical procedures such as bullectomy or lobectomy. However, surgery is associated with many complications, such as pneumothorax due to prolonged air leak, atrial fibrillation, infection, bleeding and respiratory failure, and even death in severe patients after operation [1–6].

Notably, patients with advanced age or poor cardiopulmonary functional reserves might be not ideal candidates for or may not be willing to undergo surgical procedures [7]. Although great efforts have been made to improve the safety and efficacy of surgical procedures in treating high-risk patients with GPB over the past three decades [8–11], controlling surgical damage, reducing postoperative complications, and improving therapeutic effectiveness are still challenging [12].

In this study, we introduce an alternative convenient procedure named intrabullous adhesion pexia (IBAP) involving computer tomography (CT)-guided percutaneous pulmonary bulla centesis, IBAP with biomedical fibrin sealant, and closed intracavity drainage under continuous negative pressure. We evaluated the efficacy and safety of IBAP in GPB patients who either had contraindications or were unwilling to undergo surgery.

## 2. Materials and Methods

### 2.1. Patient Recruitment and Study Design

All of the GPB patients were enrolled based on CT diagnosis and symptomatic dyspnea, and they were admitted to hospital from the outpatient department. These consecutive patients were unsuitable for or unwilling to accept surgical resection but willing to accept IBAP treatment.

According to underlying comorbidities, such as acute exacerbation of COPD, pulmonary infection, bronchial asthma, or bronchiectasis, patients received necessary treatments, such as anti-inflammatories, anti-infectives, or antihistamines, prior to IBAP procedures. Antibiotics, such as cephazolin or amikacin, were employed as preventive medication. Appropriate antibiotics were used according to the infection.

This was a prospective, nonrandomized, single-arm, unblinded study. Because of insufficient sample, the research period occurred over 13 years; all efforts were made to maintain similar procedures and technical skills of the pulmonologist during this 13-year study. All of the operative procedures were performed by the same pulmonologist and the same fibrin sealant was used in all of the operations.

The primary end point was the closure ratio of the target GPB in one month after the IBAP procedures. The secondary end points were the mortality related to the IBAP procedure, dyspnea scores, pulmonary function, 6 MWT, and the reappearance ratio of the closed GPB or the shrunk GPB in 12 months after the IBAP procedures.

This study was approved by the institutional ethics committee and conducted in compliance with the Declaration of Helsinki. Informed consent was obtained from all of the patients.

### 2.2. Operative Procedure

All of the operative procedures were performed by the pulmonologist, the professor, and the chief physician of the Department of Respiratory and Critical Care Medicine, Mingzhou Hospital, Zhejiang University. The method did not change and the professional level of the pulmonologist was stable throughout the 13 years.

Intrabullous adhesion pexia (IBAP) procedure was performed under local anesthesia in the CT scan suite, and the vital signs of patients were closely monitored during the perioperative period. Percutaneous pulmonary bulla centesis was performed by omnidirectional injection into the GPB with 10 ml of porcine fibrin sealant such that the biological glue could be distributed evenly within the target bulla. The Porcine Fibrin Sealant kit was produced by Hangzhou Puji Biotechnology Co., Ltd, containing 5 ml of 30.0 mg/ml fibrinogen in 6.0–7.0 mg/ml sodium chloride solution and 5 ml 450–850 IU/ml thrombin in 35.0–45.0 mmol/L calcium chloride solution. Then, the sealant was inserted into the target GPB through a disposable pigtail drainage catheter (14 G) attached to a closed intracavity drainage set under continuous negative pressure (at 14–18 cm H_2_O). For secondary pneumothorax caused by the bulla centesis, pleural cavity-closed drainage under continuous negative pressure (at 14–18 cm H_2_O) was a routine additional procedure. When the target GPB closed or collapsed to stable cavities without secondary pneumothorax for 72 hours with radiological examination (chest CT scan), the drainage catheters were blocked, and then would be removed 24 hours later after further confirmation.

### 2.3. Assessment

On the first day of admission and one week after the drainage tube was removed, the severity of patients' condition was evaluated according to the results of objective tests. Arterial oxygen tension (PaO_2_) and arterial carbon dioxide tension (PaCO_2_) were examined by a blood-gas analyzer (GEM Premier 3000, USA). If the patient's condition permitted, pulmonary function including forced expiratory volume in 1 second (FEV1), residual volume (RV), and total lung capacity volume (TLC) was calculated from the ﬂow-volume curve by the qualified technician using spirometry (CHEST AC-8800-D, Japan). Twelve cases in 11 patients did not complete pulmonary function tests because they were unable or unwilling to cooperate. Functional exercise capacity was assessed by using a 6 min walking distance (6 MWD) test. The 6 MWD test results of patients who could not walk initially were marked as 0 meter. The relationship between dyspnea and physical disability was evaluated by dyspnea index (from 1 to 5) based on the Medical Research Council (MRC) scale [13], which classifies this symptom into five grades (on a scale from I to V). The chest CT was performed at the 3rd month, 6th month, and 12th month after the primary end point.

### 2.4. Clinical Follow-Up

As a routine, clinical follow-up was carried out with outpatient service and chest CT scan. Outcomes assessed were closure time of the target GPB after the IBAP procedures, mortality related to the IBAP procedure, and the reappearance of the closed GPB or progress of the shrunken GPB after the IBAP procedures.

### 2.5. Statistical Analysis

All of the data were tested for normality and presented as mean ± standard deviation (SD). Differences between the preoperative and postoperative data were tested by paired Student's *t*-test using SPSS 17.0 software. A *P* value < 0.05 was considered significant.

## 3. Results

### 3.1. Patient Characteristics

A total of 38 cases in 36 patients (33 males and 3 females) with the average age of 67.53 years (range: 41–78) were diagnosed as GPB from December 2004 to April 2017 and underwent IBAP treatment. According to CT scan findings, the target GPB cavities varied in size from smallest at 10 cm × 7 cm × 5 cm to the largest cavity at 15 cm × 8 cm × 30 cm (anteroposterior diameter × medial-lateral diameter × superoinferior diameter). Moreover, there were 11 target GPBs located in the left lung and 27 in the right. Two patients had two target GPBs, while the other 34 patients each had a single target GPB. As shown in Table 1, all cases enrolled in this study had various degrees of dyspnea (four patients with grade II, 9 with grade III, four grade IV, and 21 with grade V) before IBAP treatment, and a total of 34 cases with grade III to V were classified as having serious dyspnea. Besides, 31 cases met the criteria of respiratory failure (PaO_2_ < 60 mmHg) based on arterial blood gas (ABG) analysis. All of the cases except for two cases had underlying pulmonary comorbidities, and 15 other cases were complicated by cor pulmonale. Chronic obstructive pulmonary disease (COPD) was the leading disease diagnosed in 23 cases, followed by asthma (five cases), interstitial lung disease (5 cases), and bronchiectasis (4 cases). One patient (with two GPB cases) had no comorbidities but had a history of spontaneous pneumothorax. Other co-occurring illnesses that may have influenced respiratory function or acid-base balance in blood and survival of the patients included one case each of liver cancer, esophageal cancer, rectal carcinoma and hernia of the ventral wall, prostate cancer, and bilateral hydronephrosis caused by urinary calculi.

### 3.2. Therapeutic Effectiveness

**Figure 1:**
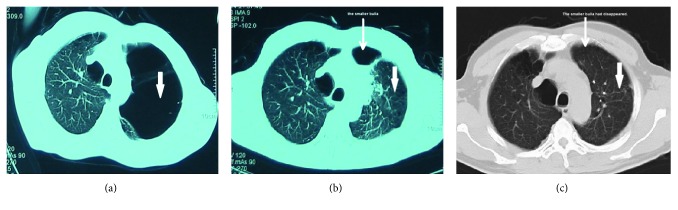
Case 3 (patient 2) of GPB treated by IBAP as shown by CT scan. The 52-year-old male patient with bilateral pulmonary bullae and left GPB (thick arrow) (a). Seven days after IBAP in July 2006, the GPB closed completely (thick arrow), while a smaller bulla in the same side appeared (thin arrow), which may be previously compressed by the target GPB (b). Six years after IBAP, the left GPB did not recur (thick arrow) and the smaller one disappeared (thin arrow) (c).

**Figure 2:**
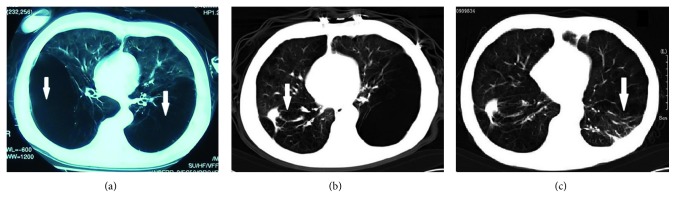
Case 8 and case 17 (patient 7) of GPB. The 65-year-old male patient, who was complicated by COPD, cor pulmonale, hernia of ventral wall, and rectal carcinoma, had two target GPBs in each side of the lung (thick arrows) (a). He accepted the first IBAP for the right GPB in February 2007 (thick arrow) (b) and the second IBAP for the left GPB in December 2008 (thick arrow) (c). No bulla recurred until he died of rectal carcinoma in March 2010.

The target GPB in 29 cases were closed within 7 days and 4 closed within 15 days after IBAP procedure. The GPB in the remaining 5 cases shrank to stable cavities in 10 days (3 cases), 14 days (1 case), and 30 days (1 case), respectively. The outcome indicated high effectiveness in closure of the target GPB, with a closure ratio of 86.84% (33/38) in one month after the IBAP procedures. The time length with chest tube in place was 9.00 ± 5.33 days in this study group. A representative case is shown in Figure 1 to visually demonstrate the therapeutic effect of the IBAP. To ensure patient safety, two patients (patient #1 and patient #7) with two separate target GPBs accepted two rounds of IBAP treatment with an interval between the two procedures. Patient #7 had comorbidities, such as COPD, cor pulmonale, rectal carcinoma, and hernia of ventral wall, and accepted IBAP for the GPB at the right lung in February 2007 and treatment for the left lung in December 2008 as shown in Figure 2. There was no recurrent bulla until the patient died of rectal carcinoma in March 2010.

After treatment, dyspnea in all of the cases was relieved or even disappeared as the index was significantly decreased from 4.11 ± 1.11 to 2.24 ± 1.15 (*P* < 0.01). Patients also showed significant improvement in pulmonary function and exercise tolerance. As shown in Table 2, mean FEV1 (L) increased from 1.06 ± 0.73 to 1.57 ± 1.13 (*P* < 0.01), while RV (L) decreased from 2.77 ± 0.54 to 2.36 ± 0.38 (*P* < 0.01) and TLC (L) decreased from the level of 6.46 ± 1.21 to 5.86 ± 1.08 (*P* < 0.01). Furthermore, PaO_2_ (mmHg) increased from 52.18 ± 8.31 to 68.29 ± 12.34 and respiratory failure was reversed in 25 of 31 patients (*P* < 0.01), while PaCO_2_ declined but without statistical significance (*P* > 0.05). The 6 MWD, which represented exercise tolerance, was also improved after treatment as the mean distance (m) walked in 6 minutes increased 129.36% from 131.58 ± 105.24 to 301.79 ± 197.90 (*P* < 0.01).

Heterogeneity was investigated by subgroup analysis. The study population was further stratified into COPD group and non-COPD group based on history of COPD before GPB treatment. As shown in Table 3, the pulmonary function and exercise tolerance improved in patients with or without history of COPD (*P* < 0.01). Importantly, significant change in the level of PaCO_2_ was observed, which decreased in the COPD group (*P* < 0.01) but increased in the non-COPD group (*P* < 0.01) after IBAP. It is worth mentioning that three patients (patient #2, 7, and 8), who could barely walk initially before IBAP treatment, had an improved 6 MWD of 42 m, 72 m, and 528 m, respectively.

### 3.3. Adverse Events

**Figure 3:**
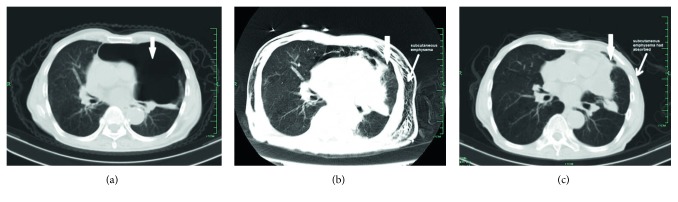
The patient 35 (case 37), a 75-year-old man, was complicated by COPD, respiratory failure, cor pulmonale, coronary heart disease, hypertension, chronic renal insufficiency, and prostate cancer, had the target GPB in left of the lung (thick arrow) (a). Fourteen days after IBAP in April 2016, the GPB closed completely (thick arrow), while obvious subcutaneous emphysema secondary to tension pneumothorax remained to be absorbed (thin arrow) (b). Three weeks later, the subcutaneous emphysema disappeared (thin arrow) (c), and no bulla recurred (thick arrow) (c) until he died of sudden cardiac death at home in February 2017.

**Figure 4:**
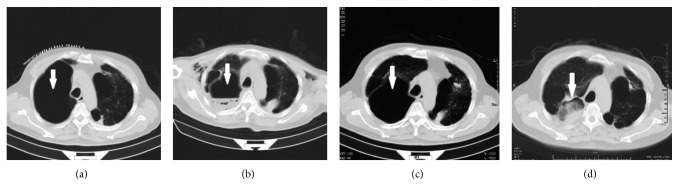
The patient 30 (case 32), a 59-year-old man, was complicated by pneumoconiosis, cor pulmonale, uarthritis, hypertension, and respiratory failure, had the GPB in the right lung (thick arrow) (a). The target GPB shrank to stable cavity in 30 days after the IBAP in March, 2013 (thick arrow) (b) until the second follow-up CT scan in April 2014 (thick arrow) (c). The right GPB closed completely observed at the third follow-up CT scan 3 years after IBAP in March 2016 (thick arrow) (d).

As shown in Table 4, the most common complications were mild noninfective inflammation of the ipsilateral lung reported in all 38 cases (Figure 1(b), Figure 3(b), and Figure 4(b)). In addition, ipsilateral pleural effusion was reported in 32 cases (31 patients) while ipsilateral secondary pneumothorax was reported in 24 cases (22 patients) as common complications. Only 5 cases (5 patients) of pleural effusion required nonroutine additional closed drainage. Ipsilateral secondary pneumothorax was absorbed gradually by pleural cavity closed drainage under continuous negative pressure as a routine additional procedure during IBAP. There were only two cases of tension pneumothorax, both of which were resolved within one month. It is shown in Figure 3 by the third representative case (patient #35) with tension pneumothorax after the IBAP procedure. Subcutaneous emphysema developed in 7 cases (6 patients), which was mild in severity and was self-absorbed later. Asymptomatic ipsilateral pleural thickening was found in 13 cases via CT scan. There were no cases of bronchopleural fistula and no deaths occurred during IBAP treatment.

### 3.4. Clinical Follow-Up

Thirty-two of 36 patients were clinically followed up until April 30, 2017, with a median follow-up time of 67.53 months (9 to 148 months), and 4 patients were lost at 10, 50, 52, and 56 month, respectively, of follow-up. Over 12 years, 14 patients died of various diseases such as acute exacerbation of COPD in six patients at month 12, 53, 58, 60, 99, and 114 after IBAP, respectively; acute coronary syndrome in four patients at month 73, 62, 26, and 10 after IBAP, respectively; esophageal cancer in one patient at the 42nd month, rectal carcinoma in one patient at the 37^th^ month, sepsis caused by trauma in one patient at the 88th month, and severe pneumonia in one patient at the 17th month after IBAP, respectively. None of these deaths was related to the IBAP treatment. At the conclusion of the follow-up deadline, 18 patients were alive and 4 of them (patient #1, 2, 5, and 30) still had normal indices at the deadline.

The thoracic CT scan showed none of GPB relapsed or aggravated in these patients during clinical follow-up. While an inspiring phenomenon was observed in patient #30, wherein the target GPB, which shrank to a stable cavity in the original IBAP procedure, had closed completely after 3 years (Figure 4).

## 4. Discussion

It is generally believed that a single small pulmonary bulla, which has negligible influence on lung functions, does not necessarily require treatment. However, for GPB or GEB, especially tension GPB, invasive therapy might be inevitable. Surgical intervention, such as bullectomy or lobectomy, may alleviate symptoms, improve exercise tolerance, and prevent fatal respiratory distress, and it is considered as the routine technique of choice in treating GPB [6, 14]. However, contraindications or complications with the open procedure hamper their clinical application in critical cases, especially for elderly patients [1, 6, 15]. Furthermore, it is highly risky to treat patients with cardiopulmonary insufficiency or other serious underlying conditions by surgical intervention. Damage to the chest wall and postoperative pain would further worsen respiratory function [16]. Therefore, minimally invasive procedures are favorable as alternative methods for the treatment of GPB. Takizawa et al. [9] reported two cases of GPB treated by CT-guided bulla drainage and showed short-term symptomatic improvement. One patient died of pulmonary dsyfunction 21 months later, while the other suffered from prolonged air leak closed with bronchoscopic bronchial occlusion later. Kemp et al. [17] reported one case of GPB who underwent bronchoscopic autologous blood installation in which the patient demonstrated slow improvement in pulmonary function and symptoms. Santini et al. [11] invented a new therapeutic strategy by installing a one-way valve in the segmental bronchi to functionally isolate the airway that supplied the bulla for surgically unfit GPB patients. Except PaO_2_ and PaCO_2_, this strategy showed a significant improvement of respiration in a relatively short-term follow-up. Thus, there is a need to further explore an alternative approach to treat GPB with affirmative safety and long-term effectiveness.

It is hypothesized that stopping one-way aerating manner of pulmonary bulla by injection of fibrin sealant into the target GPB followed by closed intracavity drainage under continuous negative pressure could achieve closure of GPB immediately. Expansion ceaselessly of pulmonary bulla can also compress the surrounding parenchyma, eventually resulting in decreased pulmonary function [18]. On the basis of mechanism of pulmonary bulla formation, an alternative method, the IBAP procedure as mentioned above, was performed under local anesthesia in this study, which achieved high effectiveness in closure of the target GPB with a closure ratio of 86.84% (33/38) in one month after the IBAP procedures, while the other 5 target GPBs shrank to stable cavities, indicating high total effective rate of 100% (38/38). Furthermore, IBAP demonstrated a good safety profile; no deaths were related to the IBAP procedure. The intentional procedure of IBAP may have broken the medical traditional taboo of percutaneous pulmonary bulla centesis. Specifically, IBAP treatment resulted in long-term effectiveness with a median follow-up time of 67.53 months (9 to 148 months) accompanied by significant improvement in exercise tolerance and pulmonary function by 6 MWD, FEV1, RV, TLC, PaO_2_, and PaCO_2_ tests (Table 2). Overall, this was a 13-year study of a minimally invasive procedure with reliable safety and encouraging long-term efficacy.

Talcum slurry has been used for decades in pleurodesis to encourage adhesion for recrudescent spontaneous pneumothorax by stimulating exuberant fibrotic response on the surface of visceral and parietal pleura, which is time consuming and may cause unfavorable complications, such as chest pain, fever, nausea, and even respiratory distress [19–21]. In comparison, the porcine fibrin sealant used in the IBAP procedure can be solidified at 37°C and produce intrabullous adhesion within a short time of 3 to 5 minutes by mediating noninfectious inflammation on the surface of the bulla wall, which allows closure of the bulla and re-expansion of collapsed lung parenchyma. Closed intracavity drainage under continuous negative pressure following IBAP can accelerate the process.

Several studies have reported restricted functional improvement after bullectomy in patients with very low FEV1, hypoxaemia, and hypercapnia before surgery [22, 23]. However, most patients in this study who had underlying diseases or poor cardiopulmonary function regarded as surgery contraindications achieved significant improvements in pulmonary function. This difference may be explained as normal or relatively normal pulmonary parenchyma that would have been resected by bullectomy or lobectomy and was reserved by the IBAP procedure. This suggests that IBAP treatment not only reduces dead space effect by closing GPB but also increases the ventilation area by releasing compressed lung tissue. This may be an advantage of IBAP compared with surgical procedures, such as bullectomy and lobectomy.

Although the overall safety profile of this study was promising, there also were complications, such as mild noninfective inflammation of the ipsilateral lung, ipsilateral pleural effusion, and ipsilateral secondary pneumothorax. Ipsilateral pleural effusion and pleural thickening were the major adverse reactions of porcine fibrin sealant, while noninfective inflammation of the ipsilateral lung might have reinforced IBAP. In consideration of the counteraction of anti-inflammation, which might have a negative influence on IBAP, glucocorticoids were not employed in the perioperative period. Ipsilateral secondary pneumothorax was the complication of percutaneous pulmonary bulla centesis. Nevertheless, most of these events were mild and of low frequency, and most were resolved. As a routine procedure and a routine additional procedure of IBAP, intrabullous catheter drainage and pleural cavity catheter drainage under continuous negative pressure might have not only facilitated target bulla closure but also alleviated noninfective inflammation, pleural effusion, pleural thickening, and secondary pneumothorax. In addition, there were five cases that did not completely close but remarkably shrank to stable cavities after IBAP procedure. We posit that broncho-bullous fistula, which stalled bulla collapse, may have played an important role in this phenomenon. It may be solved by increasing the dosage of fibrin sealant and/or increasing the frequency of IBAP procedure. Furthermore, the addition of one-way endobronchial valve placement may also shorten the IBAP procedure [24–26].

## 5. Conclusion

In conclusion, IBAP is a promising strategy for the treatment of GPB, which showed noteworthy therapeutic effect, desirable safety, and remarkable long-term efficacy. IBAP treatment will benefit a wider spectrum of symptomatic GPB patients who are unsuitable for or unwilling to accept surgical resection. However, larger studies are needed to adequately validate the efficacy and safety of IBAP for GPB.

## Figures and Tables

**Table 1:** Clinical characteristics of patients.

Case no.	Patient no.	Sex	Age	Dyspnea index	ABG analysis (mmHg)		Lung function (L)			6 MWD (m)
					PaO_2_	PaCO_2_	FEV1	RV	TLC	
1	1	M	41	3	61	38	1.95	2.20	6.45	215
2	1	M	42	2	68	40	2.37	1.87	5.35	347
3	2	M	52	5	50	30				0
4	3	M	42	5	46	34	0.59	2.76	6.60	21
5	4	M	62	3	64	34	2.39	1.89	6.33	289
6	5	M	41	3	57	45	0.45	3.58	6.98	286
7	6	M	71	5	56	53	1.10	2.85	6.92	35
8	7	M	64	5	49	40				0
9	8	F	47	5	42	31				0
10	9	M	58	5	45	50				92
11	10	F	43	2	55	36	1.70	2.57	3.61	311
12	11	F	64	5	45	30	0.45	2.91	6.45	125
13	12	M	57	2	74	35	2.18	1.96	6.33	234
14	13	M	63	5	42	67	0.41	3.45	7.51	64
15	14	M	69	5	50	50	0.46	2.82	7.15	56
16	15	M	63	5	45	31	0.40	2.54	2.85	122
17	7	M	65	4	50	36	.			97
18	16	M	61	3	58	37	0.72	3.41	7.31	296
19	17	M	48	5	46	31	.			56
20	18	M	69	5	42	62	0.39	3.25	7.20	64
21	19	M	71	5	45	39	0.63	2.76	6.40	26
22	20	M	65	3	56	53				152
23	21	M	72	5	50	38	0.41	3.21	7.62	125
24	22	M	71	5	42	65				56
25	23	M	61	5	45	52				38
26	24	M	59	3	59	40	0.60	3.65	7.60	296
27	25	M	69	5	44	57	0.43	2.62	7.18	55
28	26	M	61	2	70	32	2.40	1.84	6.33	246
29	27	M	67	3	56	46	0.70	3.47	7.21	280
30	28	M	44	5	50	37				53
31	29	M	58	3	52	39	1.05	2.24	4.15	48
32	30	M	59	4	55	33	0.89	2.55	6.92	135
33	31	M	56	5	45	41				98
34	32	M	68	3	61	45	2.22	3.02	7.55	243
35	33	M	78	5	51	57				14
36	34	M	60	5	45	37	1.08	2.88	6.11	78
37	35	M	75	4	62	46	0.99	3.12	7.02	182
38	36	M	70	4	50	56	0.63	2.67	6.87	165

M: male; F: female; IBAP: intrabullous adhesion pexy; ABG: arterial blood gas; 6 MWD: 6 min walking distance; FEV1: forced expiratory volume in 1 s; RV: residual volume; TLC: total lung capacity volume; PaO_2_: arterial partial pressure of oxygen; PaCO_2_: arterial partial pressure of carbon dioxide. 1 mmHg = 0.133 kPa. Cases 1 and 2 and cases 8 and 17 are the same patients, respectively. Twelve cases in 11 patients (patients 2, 7, 8, 9, 17, 20, 22, 23, 28, 31, and 33) did not test lung function due to their serious conditions or unwillingness. A few patients might give poor cooperation in lung function test because of their serious conditions so that a few values of pulmonary function test might be suspected in their accurateness, but this uncertainty would not affect the statistical conclusion for their own single-arm test values.

**Table 2:** Therapeutic effect of IBAP assessed by lung function, ABG analysis, 6 MWD test, and dyspnea index.

Items	*N* (cases)		Before IBAP^§^	After IBAP^§§^
Lung function (L)	26	FEV_1_	1.06 ± 0.73	1.57 ± 1.13^*∗*^
		RV	2.77 ± 0.54	2.36 ± 0.38^*∗*^
		TLC	6.46 ± 1.21	5.86 ± 1.08^*∗*^

ABG (mmHg)	38	PaO_2_	52.18 ± 8.31	68.29 ± 12.34^*∗*^
		PaCO_2_	42.71 ± 10.29	41.05 ± 4.57^#^

6 MWD (m)	38		131.58 ± 105.24	301.79 ± 197.90^*∗*^

Dyspnea index	38		4.11 ± 1.11	2.24 ± 1.15^*∗*^

Data are represented as mean ± standard deviation (SD). As shown in Table 1, 12 of 38 cases did not test lung function due to serious conditions or their unwillingness. IBAP: intrabullous adhesion pexy; ABG: arterial blood gas; 6 MWD: 6 min walking distance; FEV1: forced expiratory volume in 1 s; RV: residual volume; TLC: total lung capacity volume; PaO_2_: arterial partial pressure of oxygen; PaCO_2_: arterial partial pressure of carbon dioxide. 1 mmHg = 0.133 kPa. ^*∗*^*P* < 0.01; ^#^*P* > 0.05 (the value of posttreatment compared to before). ^§^On the first day of admission; ^§§^one week after the drainage tube was removed.

**Table 3:** Therapeutic effect in patients stratified by COPD.

Parameter	*N* (cases)	Items		Before IBAP^§^	After IBAP^§§^
COPD	15	Lung function (L)	FEV_1_	0.74 ± 0.47	1.03 ± 0.68^*∗*^
			RV	3.06 ± 0.32	2.49 ± 0.27^*∗*^
			TLC	7.04 ± 0.42	6.24 ± 0.53^*∗*^
	23	ABG (mmHg)	PaO_2_	50.09 ± 6.15	62.26 ± 7.71^*∗*^
			PaCO_2_	47.91 ± 9.82	41.70 ± 5.46^*∗*^
		6 MWD (m)		107.74 ± 92.24	263.48 ± 203.84^*∗*^
		Dyspnea index		4.43 ± 0.84	2.70 ± 1.11^*∗*^

Non-COPD	11	Lung function (L)	FEV_1_	1.49 ± 0.82	2.30 ± 1.24^*∗*^
			RV	2.38 ± 0.55	2.17 ± 0.43^*∗*^
			TLC	5.67 ± 1.50	5.33 ± 1.40^*∗*^
	15	ABG (mmHg)	PaO_2_	55.40 ± 10.25	77.53 ± 12.57^*∗*^
			PaCO_2_	34.73 ± 3.99	40.07 ± 2.60^*∗*^
		6 MWD (m)		168.13 ± 116.35	360.53 ± 179.16^*∗*^
		Dyspnea index		3.60 ± 1.30	1.53 ± 0.83^*∗*^

Data are expressed as mean ± standard deviation (SD). As shown in Table 1, 12 of 38 cases did not test lung function due to serious conditions or their unwillingness. IBAP: intrabullous adhesion pexy; ABG: arterial blood gas; 6 MWD: 6 min walking distance; FEV1: forced expiratory volume in 1 s; RV: residual volume; TLC: total lung capacity volume; PaO_2_: arterial partial pressure of oxygen; PaCO_2_: arterial partial pressure of carbon dioxide. 1 mmHg = 0.133 kPa. ^*∗*^*P* < 0.01 (the value of posttreatment compared to before). ^§^On the first day of admission; ^§§^ one week after the drainage tube was removed.

**Table 4:** The complications in perioperative period in 38 cases.

Perioperative complications	*N* (cases)	Incidence rates (%)
Noninfective inflammation of the ipsilateral lung	38	100.00
Ipsilateral pleural effusion	32	84.21
** **In a small amount	27	71.05
** **In a large amount needing drainage	5	13.16
Ipsilateral secondary pneumothorax	24	63.16
** **Nontension pneumothorax	22	57.90
** **Tension pneumothorax	2	5.26
Subcutaneous emphysema	7	18.42
Asymptomatic ipsilateral pleural thickening	13	34.21
Bronchopleural fistula	0	0.00
Mortality	0	0.00

## Data Availability

The data used to support the findings of this study are available from the corresponding author upon request.
